# *In vitro* propagation and assessment of genetic stability of acclimated plantlets of *Cornus alba* L. using RAPD and ISSR markers

**DOI:** 10.1007/s11627-016-9781-6

**Published:** 2016-08-26

**Authors:** Agnieszka Ilczuk, Ewelina Jacygrad

**Affiliations:** 1 Faculty of Horticulture, Biotechnology and Landscape Architecture, Department of Ornamental Plants, Warsaw University of Life Sciences, ul. Nowoursynowska 159, 02-766 Warsaw, Poland; 2 Department of Plant Pathology, University of California, Davis, One Shields Avenue, Davis, CA 95616-8751 USA

**Keywords:** Dogwood, Micropropagation, Molecular marker, Rooting, Survival rate

## Abstract

*Cornus alba* L. (white dogwood) is an important ornamental shrub having a wide range of applications such as reforestation programs and soil retention systems. The vegetative propagation of dogwood by cuttings may be slow, difficult, and cultivar dependent; therefore, an improved micropropagation method was developed. Nodal stem segments of *C. alba* cultivars ‘Aurea’ and ‘Elegantissima’ were cultured on media enriched with six different sources of macronutrients. Media were supplemented with either *N*^6^-benzyladenine (BA) or thidiazuron (TDZ) in combination with 1-naphthaleneacetic acid (NAA). Regardless of the cultivar, the best shoot proliferation was observed on Lloyd and McCown medium (woody plant medium (WPM)) at pH 6.2, containing 1.0 mg L^−1^ BA, 0.1 mg L^−1^ NAA, and 20–30 g L^−1^ sucrose. Rooting of regenerated shoots was achieved by an *in vitro* method when different concentrations of NAA or indole-3-butyric acid (IBA) were tested. Microcuttings were rooted for 8 wk on medium enriched with 0.25 mg L^−1^ NAA and potted into P9 containers in the greenhouse. The final survival rate of the plants after 20 wk was 80% for ‘Aurea’ and 90% for ‘Elegantissima’. Genetic stability of the micropropagated plants was confirmed by using two DNA-based molecular marker techniques. A total of 30 random amplified polymorphic DNA (RAPD) and 20 inter-simple sequence repeat (ISSR) primers resulted in 197–199 and 184–187 distinct and reproducible band classes, respectively, in ‘Aurea’ and ‘Elegantissima’ plantlets. All of the RAPD and ISSR profiles were monomorphic and comparable with the mother plant.

## Introduction

Woody plants are essential components of the natural landscape, and public and private recreation areas having a wide range of applications such as in reforestation programs and soil retention systems. Wania *et al.* (2006) affirmed that species belonging to the *Cornaceae* family, such as white dogwood (*Cornus alba* L.), should be considered in the design of urban green areas. White dogwood produces ovate or elliptic leaves 10- to 15-cm long that turn reddish in fall, and it forms a thicket of slender red stems that become bright crimson in winter. Many cultivars are generally hardy, grow well in full sunlight or thin shade, and tolerate a wide variety of soil types. Its yellow-green to green leaves are insensitive to air pollution. *C. alba* is primarily used as an ornamental and landscape plant (Field *et al.*^2001^). Vegetative propagation of *C. alba* by stem cuttings, although sometimes used, may be slow, difficult, and cultivar dependent (Pacholczak and Szydło 2010).

Propagation by tissue culture is applicable to “difficult to propagate” species and it may offer economic advantages even for some species that are considered relatively “easy to propagate.” There are several challenges when propagating woody plants by tissue culture: for example, difficult explant sterilization and phenolic component secretion causes serious problems in establishing *in vitro* cultures and further plant regeneration. Premature death of explants can be encountered as well as vitrification, chlorosis of leaves, and little or no root induction (Ilczuk *et al.*^2013^). Several studies have been carried out to optimize conditions for the regeneration and multiplication of different *Cornus* species (Lu 1984, 1985; Lattier *et al.*^2014^; Li *et al.*^2015^). Regeneration efficiency depends on the medium composition: macro- and micronutrients, type and concentration of growth regulators, and carbon source. The most commonly used media for tissue culture of dogwood are woody plant medium (WPM; Lloyd and McCown 1980) for *Cornus florida* and *Cornus mas* (Kaveriappa *et al.*^1997^; Ďurkovič 2008) and MS (Murashige and Skoog 1962) for *Cornus kousa* (Ishimaru *et al.*^1993^), and *C. alba* (Zhang and Li 2005a, b, 2010). Other media are less frequently reported to be used: Linsmaier and Skoog (1965) (LS) for *Cornus officinalis* (Ishimaru *et al.*^1998^), Schenk and Hildebrandt (1972) (SH) for *C. florida* (Trigiano *et al.*^1989^), Driver and Kuniyuki (1984) (DKW) for *Cornus wilsoniana* (Li *et al.*^2015^), and broad-leaved tree medium (BW; Sato 1991) for *C. kousa* and *Cornus capitata* (Ishimaru *et al.*^1998^; Hadziabdic 2005).

Growth of axillary or adventitious shoots is stimulated by the presence of cytokinin in the culture medium, mainly *N*^6^-benzyladenine (BA) at 0.5–1.0 mg L^−1^ (Edson *et al.*^1994^; Konôpková and Bošiaková 2013), 0.1 mg L^−1^ zeatin (Z) with 2.0 mg L^−1^ BA (Xue *et al.*^2003^), or 0.13–0.5 mg L^−1^ thidiazuron (TDZ) (Kaveriappa *et al.*^1997^; Ďurkovič 2008), all of which are typically used in combination with 0.1 mg L^−1^ 1-naphthaleneacetic acid (NAA).

Sucrose is the main source of carbon for *in vitro* culture of many plants. The addition of sucrose to the medium enhances cell proliferation and shoot regeneration (Nowak *et al.*^2004^). Carbohydrates in the form of sugars in the medium are the major source of energy and osmoticum for regenerating explants (Stavarek *et al.*^1980^; Nowak *et al.*^2004^). Sucrose concentration is a decisive factor for shoot regeneration and growth (Gibson 2000). Several authors recommend 30 g L^−1^ sucrose as the optimal concentration to guarantee a high multiplication rate in *C. nuttallii, C. florida*, and *C. wilsoniana* (Edson *et al.*^1994^; Sharma *et al.*^2005^; Li *et al.*^2015^).

The pH of the culture medium is also important, as it controls uptake by the regenerating explants of macro- and micronutrients and growth regulators (George 2008), which in turn directly affects regeneration, *i.e.*, the number and length of microshoots. The medium acidity also regulates biochemical processes occurring in plant cells. The optimal pH for the *in vitro* culture of dogwood cultivars ranges between 5.0 and 6.5. Any pH fluctuations affect uptake from the medium of NH_4_^+^ and/or NO_3_^−^ ions and the flow of protons and hydroxyl ions (Scragg 1993). In *in vitro* cultures of *C. mas*, the pH of the medium was usually maintained in the range of 5.6–6.2 (Ďurkovič 2008; Konôpková and Bošiaková 2013).

The type and concentration of natural or synthetic auxins in a medium affects the rooting of microcuttings (Marks and Simpson 2000; De Klerk 2002). Auxin is indispensable for stimulating development of root primordia, though in further developmental phases it can limit root elongation (Overvoorde *et al.*^2010^). For rooting of white dogwood (Zhang and Li 2005b, 2010) and cornelian cherry (*C. mas*) microcuttings (Ďurkovič 2008; Ďurkovič and Bukovská 2009; Feng *et al.*^2009^), the most commonly used auxin is indole-3-butyric acid (IBA) at a concentration of 0.1–1.0 mg L^−1^, while 1.0 mg L^−1^ NAA or 0.1–2.4 mg L^−1^ indole-3-acetic acid (IAA) are infrequently applied when micropropagating *C. kousa* and *C. florida* (Ďurkovič and Bukovská 2009).

Acclimation is the final but frequently most critical step in a successful micropropagation system. Micropropagated plants have often a limited amount of epicuticular waxes, poorly developed cuticle, and incorrectly functioning stomata, and their photosynthetic rate is low (Hazarika 2003; Mišalová *et al.*^2009^; Ďurkovič *et al.*^2010^). These irregularities may lead to physiological disorders, especially in transpiration, which is the main cause of poor survival of microcuttings after placing them under *ex vitro* conditions (Ziv 1995). The information available on acclimation of *Cornus* is limited. However, previous reports indicate that 60–100% of plants can acclimate to *ex vitro* conditions depending on the species (Kaveriappa *et al.*^1997^; Ishimaru *et al.*^1998^; Ďurkovič 2008; Ďurkovič and Bukovská 2009; Li *et al.*^2015^).

Micropropagation is an efficient method of clonal propagation; however, the resulting regenerants often show somaclonal variation (Larkin and Scowcroft 1981). For commercial planting, the micropropagated plants should be true-to-type with respect to the mother plant. Hence, it is important to ensure the genetic fidelity of the micropropagated plants. Molecular analysis has been used to assess the genetic fidelity of the *in vitro-*derived clones after acclimation to *ex vitro* conditions. Random amplification of polymorphic DNA (RAPD) and inter simple sequence repeat (ISSR) markers have been used in species of the family Cornaceae for detecting genetic diversity (Ercisil *et al.*^2008^; Shi *et al.*^2010^; Hassanpour *et al.*^2013^).

Development of protocols for tissue culture (including medium composition) and successful acclimation of white dogwood to *ex vitro* conditions is key for maintaining vigorous juvenile tissue and for rapid multiplication of new elite cultivars. In addition, *in vitro* propagation protocols provide a platform for further cultivar improvements through ploidy manipulation, mutation treatments, and transgenic applications.

Information on the *in vitro* propagation of *C. alba* is scarce. There are only three publications about *C. alba* and they do not cover all aspects of micropropagation (Zhang and Li 2005a, b, 2010). The aim of the present study was to investigate the influence of various macronutrient compositions, sugar concentrations, and growth regulators in the medium on the proliferation rate and growth of shoots. The rooting potential of regenerated microcuttings was also determined as well as their ability to acclimate to *ex vitro* conditions. The genetic stability of the acclimated plants was also verified.

## Materials and Methods

### Plant material and culture conditions

Plant material for tissue culture was harvested May through September from 3-yr-old shrubs of white dogwood (*C. alba* L.) cultivars ‘Aurea’ and ‘Elegantissima’ grown in a commercial nursery M.M. Kryt Młody Materiał Szkółkarski Marcin Kryt located at Wola Prażmowska in the Masovian Voivodship.

**Figure 1.**
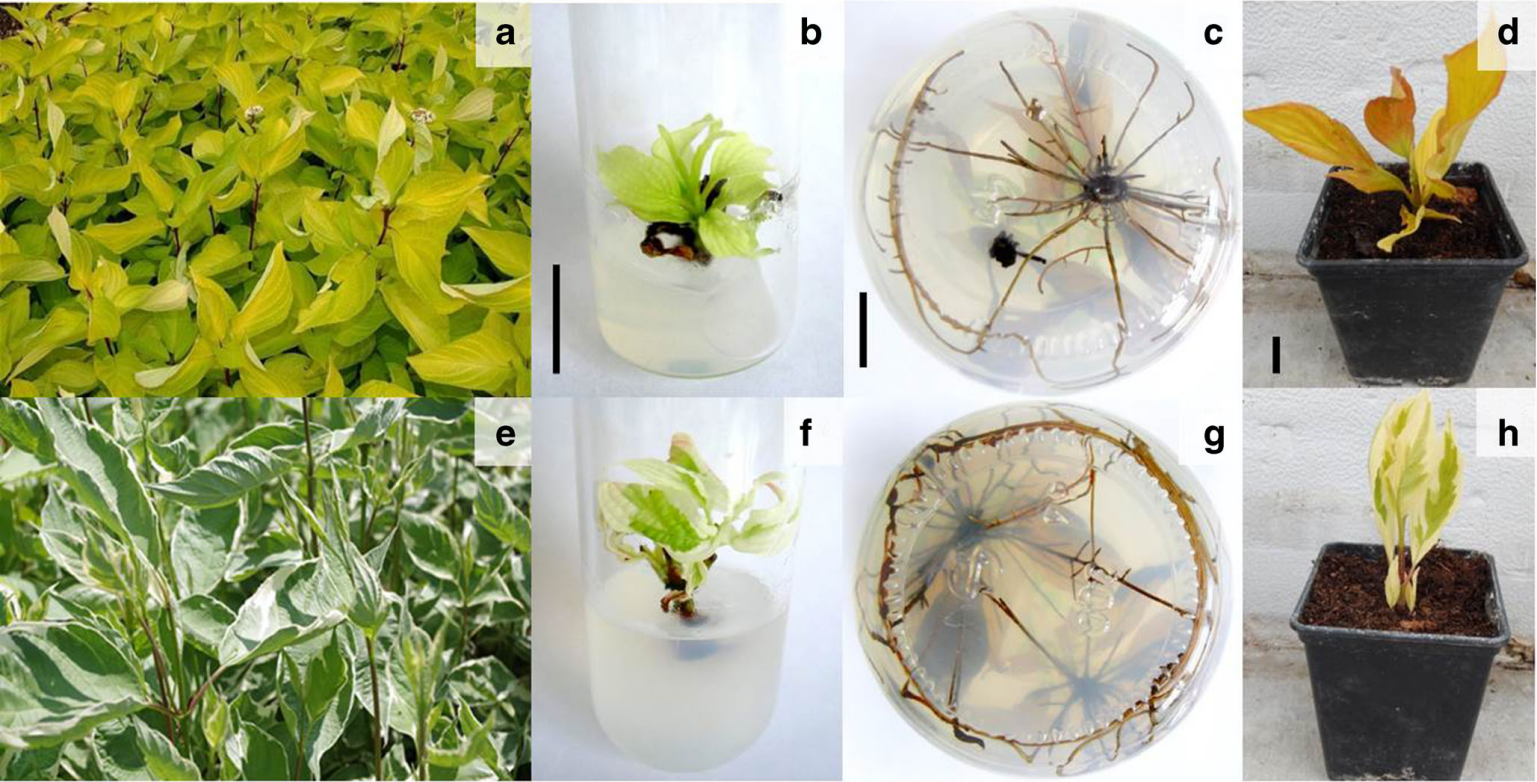
White dogwood (*Cornus alba* L.) ‘Aurea’ (*a*–*d*) and ‘Elegantissima’ (*e*–*h*). *a*, *e* Three-year-old plants grown in the summertime; *b*, *f* *in vitro* shoot proliferation on woody plant medium (WPM) with 1.0 mg L^−1^ *N* ^6^-benzyladenine (BA), 0.1 mg L^−1^ 1-naphthaleneacetic acid (NAA), and 20 g L^−1^ sucrose after 6 wk of culture; *c*, *g* rooting of shoots *in vitro* on WPM with 0.25 mg L^−1^ NAA after 8 wk of culture; *d*, *h* plantlets 2 wk after transfer to greenhouse conditions. *Bars* = 1 cm.

For culture establishment, young vegetative shoots 30 cm long were collected and washed under running tap water for 5 min to remove any surface dirt. The leaves were removed and the stems were cut into 0.8-cm nodal segments. The segments were surface sterilized with 70% (*v*/*v*) ethanol for 2 min and further sterilized with 3% (*v*/*v*) solution of sodium hypochlorite (15% NaOCl; Chempur®, Piekary Śląskie, Poland) for 15 min. After sterilization, the explants were rinsed three times in sterile distilled water for 5 min and were placed on basal WPM (Lloyd and McCown 1980) medium supplemented with 1.0 mg L^−1^*N*^6^-benzyladenine (BA; Sigma-Aldrich®) and 0.1 mg L^−1^ 1-naphthaleneacetic acid (NAA; Sigma-Aldrich®). Every 2 wk, explants were transferred onto fresh basal WPM with BA and NAA because the medium in contact with the explants had darkened. The microshoots thus obtained were the plant material that served to establish the next experiment (Fig. 1*b*, *f*).

All subsequent experimental media contained MS micronutrients and vitamins (Murashige and Skoog 1962), 20 g L^−1^ sucrose (Carl Roth®, Karlsruhe, Germany; except the fourth experiment), and 8.0 g L^−1^ Bacto™ Agar (Becton, Dickinson and Company, Sparks, MD). The pH was adjusted to 5.8 (except the third experiment) with 1 N NaOH and 1 N HCl before autoclaving at 121°C at 110 kPa for 20 min.

All macronutrients used for media preparation were obtained from POCH S.A. (Gliwice, Poland), while micronutrients and vitamins were obtained from Sigma-Aldrich®.

All the cultures were maintained in a lighted growth chamber at 23 ± 1°C with a 16/8 h light/dark cycle. The light intensity was 35 μmol m^−2^ s^−1^ from cool-white fluorescent tubes (Philips MASTER TL-D Super 80 36W/840, Philips Lighting, Eidhoven, the Netherlands).

### Effect of medium composition on multiplication rate

The first experiment compared the composition of macroelements on shoot regeneration. Shoot tips (≤1.5 cm long) coming from the established *in vitro* culture were cultured on media containing six different sources of macronutrients: AN (Anderson 1980), MS (Murashige and Skoog 1962), NN (Nitsch and Nitsch 1969), QL (Quoirin and Lepoivre 1977), SH (Schenk and Hildebrandt 1972), and WPM (Lloyd and McCown 1980). All media were supplemented with 1.0 mg L^−1^ BA and 0.1 mg L^−1^ NAA.

The second experiment evaluated axillary and adventitious shoot regeneration and elongation on WPM supplemented with 0.1 mg L^−1^ NAA alone or in combination with either BA or TDZ at (0.5, 1.0, 2.0, or 3.0 mg L^−1^). The control treatment was WPM without plant growth regulators.

The third experiment compared the effect of medium pH on shoot proliferation. Uniformly sized shoot tips (1.5 cm) were transferred to WPM containing 1.0 mg L^−1^ BA and 0.1 mg L^−1^ NAA and adjusted prior to autoclaving to various pH values (5.8, 6.2, and 6.8).

The last experiment compared the effect of various sucrose concentrations on shoot multiplication rate. Apical shoot tips (1.5 cm) were placed on WPM with 1.0 mg L^−1^ BA and 0.1 mg L^−1^ NAA supplemented with 0 (control), 10, 20, 30, 40, or 50 g L^−1^ sucrose.

After 8 wk of culture, the percentage of regenerated explants, number of shoots per explant, and shoot length were evaluated.

### Effect of auxins on rooting of microcuttings

For rooting studies, microshoots (2–3 cm long) were selected and cut midway in the internodal regions. To compare the effect of auxin type and concentration on rooting of microshoots, the microshoots were cultured on WPM supplemented with NAA or IBA at various concentrations (0.25, 0.5, and 1.0 mg L^−1^). The microshoots cultured on WPM without auxins served as the control treatment. After 8 wk from the beginning of the rooting experiment, the percentage of rooted microcuttings, root number per shoot, root length, and plant height were evaluated.

### Plant acclimation to *ex vitro* conditions

Plantlets were rooted on WPM with 0.25 mg L^−1^ NAA for 8 wk. The rooted plantlets were removed from the culture containers, and the agar medium was removed by thoroughly rinsing the root system in distilled water. The plantlets were then potted into P9 containers (Interplast Plastic Products Sp. z o. o. (Ltd), Bytom, Poland) filled with substrate composed of peat mixed with perlite in a 2:1 (*w/w*) ratio and placed into transparent plastic containers. The plantlets were sprayed with 0.1% (*w*/*v*) Proplant 722 SL (propamocarb; Agriphar S.A., Ougrée, Belgium), and then placed in the plastic containers that were covered with glass and located in a growth chamber at 24 ± 1°C with 70% relative humidity and 24 μmol m^−2^ s^−1^ light intensity using cool-white fluorescent tubes (Philips MASTER TL-D Super 80 36W/840, Philips Lighting) with a 16-h photoperiod. After 1 mo, the pots were removed from the containers and transferred to a greenhouse with a shade system at 25/19 ± 1°C (day/night) with a quantum irradiance of 180 μmol m^−2^ s^−1^. During the experiment, plant height was measured every wk after potting. Twenty weeks after potting, the percentage of plants acclimatized to *ex vitro* conditions was determined.

### Genetic stability of acclimated plants

Fresh young leaf samples were collected from a mother plant (a field-grown plant used as an explant source for culture initiation) and from 10 randomly selected 20-wk-old acclimated plants. Young leaf tissue (100 mg) was ground to a fine powder in liquid nitrogen and kept in 1.5-mL centrifuge tubes in a −80°C freezer. Genomic DNA was extracted from leaf tissue using a GeneMATRIX™ Plant and Fungi DNA Purification Kit (EUR_X_® Molecular Biology Products, Gdańsk, Poland). DNA quantity and quality were estimated using a NanoDrop™ 2000 spectrophotometer (Thermo Science™, Wilmington, DE). DNA samples were diluted to 10 ng μL^−1^ for both RAPD and ISSR reactions.

### RAPD analysis

Polymerase chain reaction (PCR) was performed in a volume of 25 μL containing 10 ng template DNA, 2.5 μL 10× *Taq* buffer with KCl, 200 μM of each dNTP, 1.5 mM MgCl_2_, 0.8 μM primer, and 0.125 U *Taq* DNA Polymerase (EUR_X_® Molecular Biology Products, Gdańsk, Poland). A total of 49 arbitrary RAPD primers (Operon Technologies, Alameda, CA) were tested for PCR amplification. Thirty of them (Table 1) were chosen for the analysis because they produced highly readable and reproducible bands. The reaction was cycled 35 times at 94°C for 40 s for denaturation of template DNA, 35°C for 1 min for primer annealing, and 72°C for 2 min for primer extension in a thermal cycler (Mastercycler® ep gradient; Eppendorf, Hamburg, Germany). The final extension cycle allowed an additional incubation for 10 min at 72°C. The samples were stored at 4°C until analysis was carried out.

**Table 1.** Primer sequences and number of scored monomorphic bands produced by RAPD markers in *Cornus alba* cultivars

Marker	Sequence (5′ – 3′)	Number of bands scored per plant	
		‘Aurea’	‘Elegantissima’
OPA 02	CAGGCCCTTC	6	5
OPA 03	AGTCAGCCAC	5	5
OPA 04	AATCGGGCTG	7	8
OPA 05	AGGGGTCTTG	9	9
OPA 06	GGTCCCTGAC	3	3
OPA 07	GAAACGGGTG	8	8
OPA 08	GTGACGTAGG	7	7
OPA 09	GGGTAACGCC	4	4
OPA 11	CAATCGCCGT	5	6
OPA 12	TCGGCGATAG	8	8
OPA 14	TCTGTGCTGG	9	8
OPA 15	TTCCGAACCC	9	9
OPA 16	AGCCAGCGAA	7	7
OPA 17	GACCGCTTGT	7	6
OPA 18	AGGTGACCGT	8	8
OPA 19	CAAACGTCGG	6	5
OPA 20	GTTGCGATCC	9	9
OPB 01	GTTTCGCTCC	5	5
OPB 10	CTGCTGGGAC	7	8
OPC 18	TGAGTGGGTG	5	5
OPD 15	CATCCGTGCT	4	4
OPE 16	GGTGACTGTG	6	6
OPE 17	CTACTGCCGT	7	8
OPF 05	CCGAATTCCC	6	7
OPF 09	CCAAGCTTCC	8	9
OPF 10	GGAAGCTTGG	5	5
OPF 20	GGTCTAGAGG	9	8
OPH 16	TCTCAGCTGG	4	4
OPH 17	CACTCTCCTC	7	7
OPH 18	GAATCGGCCA	7	8
Total		197	199
Average number per primer		6.57	6.63

### ISSR analysis

For ISSR amplification, 34 microsatellite primers (UBC Set#9, University of British Columbia, Vancouver, Canada) were randomly selected and tested. Twenty of them (Table 2) were then chosen for the analysis because they produced highly readable and reproducible bands. PCR for ISSR amplification was performed in a volume of 25 μL with the same concentrations and volumes of reaction components as for RAPD analysis. The PCR program consisted of an initial denaturation for 5 min at 94°C, followed by 35 cycles of 45 s denaturation at 94°C, 1 min annealing at temperature shown in Table 2, and 2 min extension at 72°C, with a final extension at 72°C for 7 min. The samples were stored at 4°C until analysis was carried out.

**Table 2.** Primer sequences and number of scored monomorphic bands produced by ISSR markers in *Cornus alba* cultivars

Marker	Sequence (5′ – 3′)	Annealing temperature (°C)	Number of bands scored per plant	
			‘Aurea’	‘Elegantissima’
UBC 807	(AG)_8_T	44.6	9	9
UBC 809	(AG)_8_G	47.1	11	10
UBC 810	(GA)_8_T	44.6	10	11
UBC 811	(GA)_8_C	47.1	10	11
UBC 812	(GA)_8_A	44.6	8	9
UBC 815	(CT)8G	44.6	8	8
UBC 816	(CA)_8_T	44.6	9	9
UBC 817	(CA)_8_A	44.6	10	10
UBC 818	(CA)_8_G	47.1	7	8
UBC 823	(TC)_8_C	46.1	11	11
UBC 824	(TC)_8_G	46.1	9	8
UBC 825	(AC)_8_T	46.1	11	10
UBC 827	(AC)_8_G	52.0	10	11
UBC 835	(AG)_8_YC	50.3	8	9
UBC 836	(AG)_8_YA	48.0	11	11
UBC 840	(GA)_8_YT	48.0	9	9
UBC 841	(GA)_8_YC	48.0	7	8
UBC 842	(GA)_8_YG	50.3	10	10
UBC 848	(CA)_8_RG	50.3	9	7
UBC 890	VHV(TG)_7_	50.3	7	8
Total			184	187
Average number per primer			9.25	9.35

*Y* C/T, *R* A/G, *H* non-G, *V* non-T

RAPD and ISSR amplifications were performed at least three times and only the reproducible PCR products were scored. The amplification products for all samples were resolved on 1.5% (*w/v*) agarose basica LE (Prona®, ABO Sp. z o.o (Ltd), Gdańsk, Poland) gel using 1× TAE buffer and stained with ethidium bromide (Sigma-Aldrich®). Bands were visualized using a gel documentation system (Kodak Gel Logic 100 Digital Imaging System). The size of each amplicon was estimated by comparing with the GeneRuler™ 100 bp Plus DNA ladder (Thermo Scientific®, Waltham, MA).

### Experimental design and statistics

The experiments were conducted in a completely randomized design. There were 60 explants/microcuttings in each experiment (3 replications, each containing 20 samples). To compare the means, regenerations rate percentages were transformed according to Bliss (Wójcik and Laudański 1989). The results were subjected to analysis of variance using SPSS. Multiple comparisons among means were done using the Duncan test at *p* ≤ 0.05.

## Results

### Effect of medium composition on multiplication rate

The macroelement composition of the culture medium significantly affected regeneration from shoot tips of both dogwood cultivars (Table 3). The highest regeneration percentage in both cultivars was on WPM while the lowest was on NN and SH. With ‘Aurea’ no significant difference in regeneration percentage was found between AN and NN media, and the lowest was obtained with SH medium. With ‘Elegantissima’ the highest regeneration percentage was observed on WPM while on MS, QL, and AN the percentage of regenerating explants ranged between 82% and 78%. The regeneration percentage was significantly lower on NN and SH.

**Table 3.** The effect of macronutrient composition on shoot differentiation of *Cornus alba* cultivars

Type of medium	‘Aurea’			‘Elegantissima’		
	Regeneration rate (%)	No. of shoots per explant	Length of shoots (cm)	Regeneration rate (%)	No. of shoots per explant	Length of shoots (cm)
AN	68.3 ± 1.0^b^	3.1 ± 0.3^b^	3.9 ± 0.5^b^	78.3 ± 2.3^b^	3.4 ± 0.5^b^	4.1 ± 0.3^b^
MS	78.3 ± 1.7^c^	4.5 ± 1.1^c^	5.1 ± 0.3^c^	81.6 ± 1.9^b^	4.6 ± 0.1^c^	5.3 ± 0.5^c^
NN	63.3 ± 3.4^ab^	3.5 ± 0.8^b^	3.8 ± 0.4^b^	66.6 ± 1.6^a^	3.8 ± 0.3^b^	4.8 ± 0.2^c^
SH	58.3 ± 3.2^a^	0.8 ± 0.3^a^	2.5 ± 0.6^a^	63.3 ± 0.7^a^	0.6 ± 0.2^a^	2.5 ± 0.4^a^
QL	80.0 ± 1.9^c^	4.3 ± 0.4^c^	3.5 ± 0.4^ab^	80.0 ± 4.0^b^	4.2 ± 0.7^c^	3.8 ± 0.5^b^
WPM	95.0 ± 7.6^d^	5.7 ± 0.5^d^	6.4 ± 0.7^d^	91.6 ± 1.6^c^	6.5 ± 0.4^d^	6.2 ± 0.2^d^
Mean	73.9	3.7	4.2	76.9	3.9	4.5

Means (±SD) within a *column* followed by the same *letter* are not significantly different at *p* ≤ 0.05

*AN* Anderson (1980), *MS* Murashige and Skoog (1962), *NN* Nitsch and Nitsch (1969), *SH* Schenk and Hildebrandt (1972), *QL* Quoirin and Lepoivre (1977), *WPM* woody plant medium (Lloyd and McCown 1980)

The highest multiplication rate was observed for explants on WPM, on which an average of 5.7 and 6.5 shoots over 6 cm long formed per explant in ‘Aurea’ and ‘Elegantissima’, respectively (Table 3). ‘Elegantissima’ microshoots grew to 4–5 cm in length when cultured on AN, MS, and NN (Table 3). Significantly shorter shoots (2.5 cm) were produced from both cultivars on SH medium.

The effects of growth regulators on the percentage of regenerating explants, number of microshoots per explant, and shoot length were significant in both white dogwood cultivars (Table 4). For ‘Aurea’ the highest percentage of regenerating explants (92%) was found on the medium enriched with 1.0 mg L^−1^ BA and 0.1 mg L^−1^ NAA. A similar result was obtained for ‘Elegantissima’ with this medium and on medium supplemented with 1.0 mg L^−1^ TDZ and 0.1 mg L^−1^ NAA. Significantly, fewer explants from both cultivars were regenerated on media with higher concentrations of BA and TDZ or without any growth regulators.

**Table 4.** The effect of plant growth regulator concentrations on shoot regeneration of *Cornus alba* cultivars on WPM

Plant growth regulators (mg L^−1^)			‘Aurea’			‘Elegantissima’		
			Regeneration rate (%)	No. of shoots per explant	Length of shoots (cm)	Regeneration rate (%)	No. of shoots per explant	Length of shoots (cm)
BA	TDZ	NAA						
0	0	0	70.0 ± 3.6^a^	1.2 ± 0.2^a^	4.5 ± 0.7^b^	70.0 ± 3.6^a^	1.2 ± 0.1^a^	4.5 ± 0.6^b^
0	0	0.1	72.3 ± 5.5^a^	1.8 ± 0.6^a^	5.6 ± 0.2^c^	70.0 ± 3.6^a^	1.8 ± 0.7^a^	5.6 ± 0.1^c^
0.5	0	0.1	82.3 ± 2.4^b^	3.4 ± 0.4^b^	5.7 ± 0.7^c^	81.6 ± 1.2^b^	4.1 ± 0.9^b^	5.7 ± 0.6^c^
1.0	0	0.1	91.6 ± 1.2^d^	5.8 ± 0.9^d^	6.1 ± 1.1^d^	91.6 ± 1.2^d^	6.1 ± 0.4^d^	6.1 ± 0.2^d^
2.0	0	0.1	80.0 ± 1.9^b^	4.9 ± 0.1^c^	5.9 ± 0.5^d^	80.0 ± 5.0^b^	5.1 ± 0.9^c^	6.1 ± 0.6^d^
3.0	0	0.1	76.3 ± 2.0^a^	4.5 ± 0.8^c^	4.8 ± 1.5^b^	76.8 ± 4.0^a^	4.5 ± 0.5^c^	4.8 ± 0.2^b^
0	0.5	0.1	85.0 ± 2.5^c^	5.5 ± 0.7^d^	5.4 ± 0.4^c^	85.0 ± 4.0^c^	6.5 ± 0.5^d^	6.4 ± 0.4^d^
0	1.0	0.1	80.0 ± 1.9^b^	5.1 ± 1.0^d^	4.9 ± 0.9^b^c	91.6 ± 2.4^d^	6.4 ± 0.4^d^	6.5 ± 0.4^d^
0	2.0	0.1	79.1 ± 1.1^ab^	4.7 ± 0.5^c^	4.7 ± 0.8^b^	79.8 ± 1.8^ab^	4.8 ± 0.8^c^	4.7 ± 0.5^b^
0	3.0	0.1	78.6 ± 2.2^a^	3.9 ± 0.2^b^	2.5 ± 0.4^a^	78.3 ± 0.7^a^	4.0 ± 0.6^b^	2.5 ± 0.3^a^
Mean			79.5	4.1	5.0	80.5	4.5	5.3

Means (±SD) within a *column* followed by the same *letter* are not significantly different at *p* ≤ 0.05

*BA N* ^6^-benzyladenine, *TDZ* thidiazuron*, NAA* 1-naphthaleneacetic acid, *WPM* woody plant medium (Lloyd and McCown 1980)

The highest shoot number per explant (over 5) was formed on medium enriched with either 1.0 mg L^−1^ BA plus 0.1 mg L^−1^ NAA or 0.5–1.0 mg L^−1^ TDZ plus 0.1 mg L^−1^ NAA. The longest microshoots were produced on media containing 1.0–2.0 mg L^−1^ BA plus 0.1 mg L^−1^ NAA (both cultivars) or with 0.5–1.0 mg L^−1^ TDZ plus 0.1 mg L^−1^ NAA (‘Elegantissima’) and the shortest (2.5 cm) were produced on medium containing 3.0 mg L^−1^ TDZ plus 0.1 mg L^−1^ NAA (both cultivars).

There was no significant effect of medium pH on the percentage of regenerating explants of either cultivar. However, significant differences occurred in the number of regenerating shoots per explant (Table 5). The highest pH (6.8) reduced the multiplication ratio (4.6 shoots/explant) and the shoot length (4.5 cm), while pH range 5.8–6.2 produced significantly more shoots per explant (5.5–6.7 shoots/explant) and significantly longer shoots (5.7–6.5 cm).

**Table 5.** The effect of pH on shoot regeneration of *Cornus alba* cultivars on WPM

pH	‘Aurea’			‘Elegantissima’		
	Regeneration rate (%)	No. of shoots per explant	Length of shoots (cm)	Regeneration rate (%)	No. of shoots per explant	Length of shoots (cm)
5.8	86.0 ± 3.1^a^	5.5 ± 0.8^b^	5.8 ± 0.3^b^	81.6 ± 3.0^a^	6.5 ± 0.3^b^	5.7 ± 0.4^b^
6.2	89.0 ± 3.0^a^	5.7 ± 0.6^b^	6.5 ± 0.6^c^	86.0 ± 1.6^a^	6.7 ± 0.4^b^	6.2 ± 0.3^c^
6.8	83.3 ± 0.7^a^	4.6 ± 0.8^a^	4.5 ± 0.7^a^	83.3 ± 3.7^a^	4.6 ± 0.4^a^	4.5 ± 0.6^a^
Mean	86.1	5.3	5.6	83.6	5.9	5.5

Means (±SD) within a *column* followed by the same *letter* are not significantly different at *p* ≤ 0.05

*WPM* woody plant medium (Lloyd and McCown 1980)

In both cultivars, sucrose concentration significantly affected the percentage of regenerating explants, shoot number per explant, and shoot length (Table 6). The regeneration percentage was the highest on the medium containing 20–30 g L^−1^ sucrose, where over 90% of the explants regenerated, while the lowest percentage was found on the medium without sucrose. The above concentrations were also most suitable for shoot multiplication, producing about 6 shoots per explant, and shoots were significantly longer than those from the other treatments. The shortest shoots (1.1–1.9 cm) were those regenerated on the medium without sucrose (control treatment) for both cultivars or with 50 g L^−1^ sucrose (‘Aurea’). The shoots in ‘Elegantissima’ were significantly longer, up to 2.5 cm on the medium containing 10 or 50 g L^−1^ sucrose.

**Table 6.** The effect of sucrose concentration on shoot regeneration of *Cornus alba* cultivars on WPM

Sucrose (g L^−1^)	‘Aurea’			‘Elegantissima’		
	Regeneration rate (%)	No. of shoots per explant	Length of shoots (cm)	Regeneration rate (%)	No. of shoots per explant	Length of shoots (cm)
0	33.3 ± 2.5^a^	1.2 ± 0.2^a^	1.1 ± 0.2^a^	33.3 ± 1.3^a^	1.1 ± 0.3^a^	1.1 ± 0.2^a^
10	65.0 ± 2.0^b^	3.0 ± 0.3^b^	2.7 ± 0.4^b^	65.0 ± 3.0^b^	3.0 ± 0.1^b^	2.5 ± 0.4^b^
20	96.7 ± 1.5^d^	5.7 ± 0.7^d^	5.9 ± 0.8^d^	96.7 ± 1.9^d^	6.1 ± 0.6^d^	6.1 ± 0.2^d^
30	98.3 ± 1.5^d^	6.1 ± 0.2^d^	6.1 ± 0.7^d^	95.0 ± 1.0^d^	6.3 ± 0.5^d^	6.5 ± 0.1^d^
40	86.0 ± 1.0^c^	4.8 ± 0.3^c^	3.9 ± 0.4^c^	81.6 ± 1.4^c^	4.9 ± 0.2^c^	4.1 ± 0.2^c^
50	83.3 ± 2.7^c^	4.2 ± 0.3^c^	1.9 ± 0.6^a^	80.0 ± 1.0^c^	4.2 ± 0.2^c^	2.0 ± 0.5^b^
Mean	77.1	4.2	3.6	75.3	4.3	3.7

Means (±SD) within a *column* followed by the same *letter* are not significantly different at *p* ≤ 0.05

*WPM* woody plant medium (Lloyd and McCown 1980)

### Effect of auxin on rooting of microcuttings

Auxin type and concentration significantly affected root formation in microcuttings (Table 7). In both cultivars, the lowest NAA concentration (0.25 mg L^−1^) was the most favorable for rhizogenesis: ‘Aurea’ rooted at a frequency of 95% while ‘Elegantissima’ rooted at nearly 100%. The highest root number per shoot in both cultivars was on medium without auxin and on that enriched with 0.25 mg L^−1^ NAA. The longest roots were 11.5 and 10.5 cm for ‘Aurea’ and ‘Elegantissima’, respectively (Fig. 1*c*, *g*). Significantly, lower root numbers for both cultivars were obtained with cuttings cultured on media containing IBA, the lowest at 0.5–1.0 mg L^−1^ IBA. The higher the IBA concentration, the shorter the roots, which reached only 7.4 and 6.4 cm for ‘Aurea’ and ‘Elegantissima’, respectively, at 1.0 mg L^−1^ IBA. The medium without auxin and that supplemented with 0.25 mg L^−1^ NAA alone produced roots that were significantly longer than in any other treatment, regardless of cultivar.

**Table 7.** The effect of auxin type and concentration on rooting of microcuttings of *Cornus alba* cultivars on WPM

Plant growth regulators (mg L^−1^)		‘Aurea’				‘Elegantissima’			
		Rooting rate (%)	No. of roots per shoot	Root length (cm)	Shoot length (cm)	Rooting rate (%)	No. of roots per shoot	Root length (cm)	Shoot length (cm)
NAA	IBA								
0	0	86.6 ± 1.7^c^	5.0 ± 0.2^c^	10.4 ± 0.6^c^	8.9 ± 1.2^b^	86.6 ± 0.7^c^	5.8 ± 0.3^c^	9.4 ± 0.6^c^	7.5 ± 0.8^b^
0.25	0	95.0 ± 3.0^d^	5.0 ± 0.5^c^	11.5 ± 0.8^d^	8.5 ± 0.8^b^	98.0 ± 2.0 e	5.5 ± 0.2^c^	10.5 ± 0.8^d^	7.9 ± 0.8^b^
0.5	0	92.0 ± 2.0^d^	4.4 ± 0.2^b^	9.8 ± 0.7^c^	7.2 ± 0.9^a^	91.0 ± 5.0^d^	4.3 ± 0.4^b^	9.8 ± 0.5^c^	6.2 ± 0.3^a^
1.0	0	79.9 ± 4.7^c^	4.1 ± 0.2^b^	10.1 ± 1.4^c^	7.1 ± 1.3^a^	78.9 ± 1.1^c^	4.1 ± 0.2^b^	9.1 ± 0.6^c^	6.1 ± 0.4^a^
0	0.25	75.0 ± 5.0^b^	4.3 ± 0.6^b^	8.5 ± 0.7^b^	7.4 ± 0.3^a^	76.3 ± 1.7^b^	4.3 ± 0.3^b^	8.5 ± 0.9^b^	6.4 ± 0.3^a^
0	0.5	73.3 ± 2.3^b^	3.8 ± 0.3^a^	8.0 ± 1.5^b^	7.2 ± 0.3^a^	73.3 ± 2.5^b^	3.1 ± 0.4^a^	7.0 ± 0.4^b^	6.2 ± 0.3^a^
0	1.0	66.6 ± 2.7^a^	3.4 ± 0.1^a^	7.4 ± 0.9^a^	7.6 ± 0.3^a^	66.6 ± 4.7^a^	3.2 ± 0.4^a^	6.4 ± 0.2^a^	6.6 ± 0.5^a^
Mean		81.2	4.3	9.4	7.7	81.5	4.3	8.7	6.7

Means (±SD) within a *column* followed by the same *letter* are not significantly different at *p* ≤ 0.05

*NAA* 1-naphthaleneacetic acid, *IBA* indole-3-butyric acid, *WPM* woody plant medium (Lloyd and McCown 1980)

### Plantlet acclimation to *ex vitro* conditions

**Figure 2.**
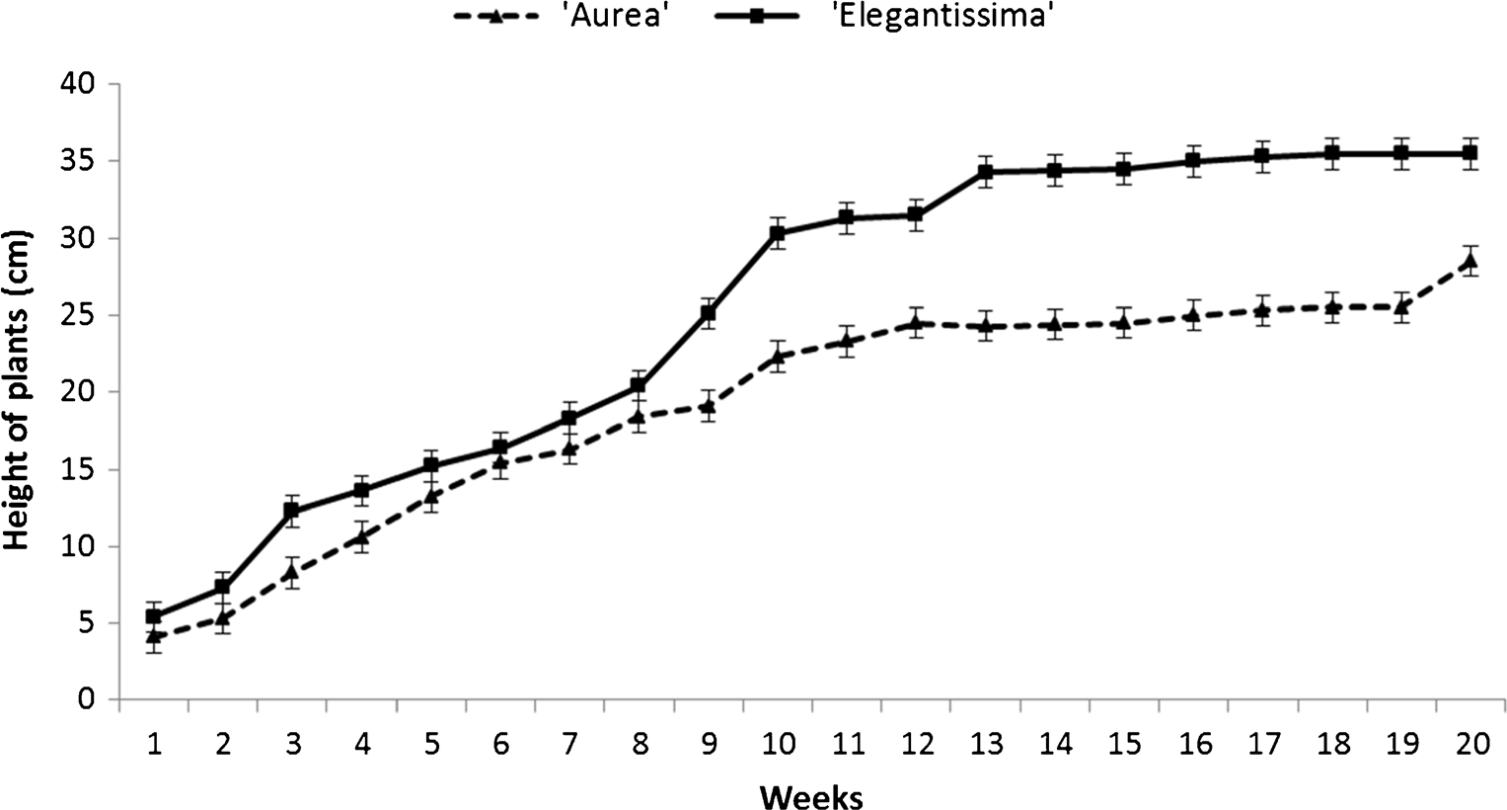
Growth dynamics of *Cornus alba* ‘Aurea’ and ‘Elegantissima’ after potting. *Error bars* indicate standard deviation.

Plantlets acclimated to greenhouse conditions at rates of 80% and 90% for ‘Aurea’ and ‘Elegantissima’, respectively (Fig. 1*d*, *h*). In growth dynamics, the differences between the two cultivars were small (Fig. 2). The most intensive growth occurred between the 2nd and 11th weeks, while between the 12th and 20th weeks the growth was slower. The plants reached final heights of approximately 28 and 35 cm for ‘Aurea’ and ‘Elegantissima’, respectively.

### Genetic stability of acclimated plants

**Figure 3.**
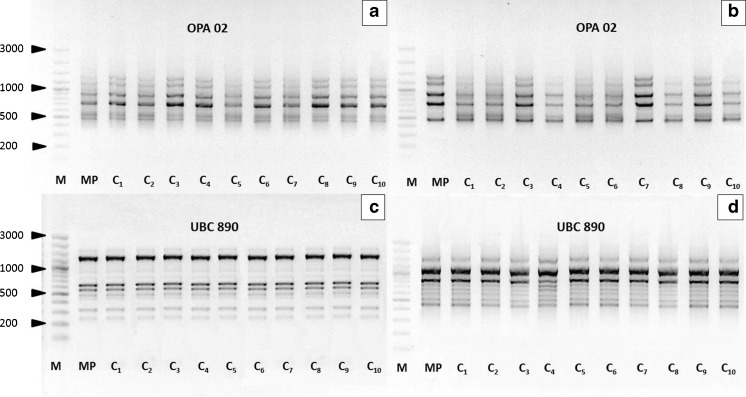
RAPD (*a*, *b*) and ISSR (*c*, *d*) profiles of plantlets of *Cornus alba* ‘Aurea’ (*a*, *c*) and ‘Elegantissima’ (*b*, *d*). *Lane M* DNA marker (100–3000 bp DNA ladder), *MP* mother plant, *C*_*1*_–*C*_*10*_ acclimated plantlets.

Analysis with RAPD markers gave a total of 197 and 199 monomorphic bands for ‘Aurea’ and ‘Elegantissima’, respectively, while with ISSR markers, 184 and 187 bands were produced for ‘Aurea’ and ‘Elegantissima’, respectively. In none of the analyzed cultivars, polymorphic bands were obtained. On average, a single RAPD marker generated 6.57 monomorphic bands for ‘Aurea’ and 6.63 for ‘Elegantissima’, ranged from 200 to 2500 bp in length (Table 1). The ISSR markers each generated an average of 9.25 and 9.35 bands for ‘Aurea’ and ‘Elegantissima’, respectively, and their lengths ranged between 250 and 2500 bp (Table 2). Using RAPD and ISSR markers, no DNA polymorphism was found between the mother plant and ten clones (C_1_–C_10_) acclimated to *ex vitro* conditions (Fig. 3).

## Discussion

Micropropagation of woody plants may be difficult and its success depends on the culture medium composition, especially macronutrients. Among several culture media tested on *C. alba* ‘Aurea’ and ‘Elegantissima’, WPM produced both the highest regeneration rate and the highest shoot number per explant. Similar results were obtained in *C. nuttallii* (Edson *et al.*^1994^), *C. officinalis* (Xue *et al.*^2003^), *C. mas* (Ďurkovič 2008), and *C. florida* (Kaveriappa *et al.*^1997^; Konôpková and Bošiaková 2013). *C*. *kousa* regenerated well on WPM (Konôpková and Bošiaková 2013), ½ BW (broad-leaved tree medium; Sato 1991) (Hadziabdic 2005), and MS (Ishimaru *et al.*^1993^). WPM has ion concentrations similar to those of AN and NN while containing half those of MS. The nitrogen content of WPM is relatively low (^1^/_4_ of that in MS) but comparable to that in AN while the sulfate level is 5-fold that in MS and AN and the potassium concentration is ^2^/_3_ that in MS and QL. Shoot cultures of woody plants may be more efficient in uptake of N and K such that lower levels of these elements in the tissue culture medium are sufficient for shoot growth and proliferation.

The presence of growth regulators in a culture medium is indispensable for normal regeneration of explants and growth of microshoots. Enrichment of medium with BA was favorable for shoot formation in *C. nuttallii* (Edson *et al.*^1994^), *C. florida* (Declerk and Korban 1994; Kaveriappa *et al.*^1997^), *C. kousa* (Hadziabdic 2005), and *C. mas* ‘Titus’ (Lech *et al.*^2009^). However, *C. mas* ‘Devin’ (Lech *et al.*^2009^) needed supplementation of NAA at a concentration of 0.1 mg L^−1^. In the present study, a similar response was observed in *C. alba*: both cultivars formed shoots on medium containing 1.0 mg L^−1^ BA and 0.1 mg L^−1^ NAA. However, Zhang and Li (2005b, 2010) found that the most suitable concentration for *C. alba* overall was 0.5 mg L^−1^ BA and 0.3 mg L^−1^ IBA, while medium containing 0.5 mg L^−1^ BA and 0.05 mg L^−1^ IBA was better for ‘Aurea’ in particular. According to Ďurkovič (2008), for cornelian cherry ‘Macrocarpa’, the combination of 0.7 mg L^−1^ BA and 0.05 mg L^−1^ NAA was optimal and resulted in the highest number of regenerating shoots. Edson *et al.* (1994) propagated *C. nuttallii* ‘Ascona’ and obtained the highest shoot number in the presence of 1.0 mg L^−1^ BA in the culture medium. For *C. florida* the best results were obtained on medium containing 0.5 mg L^−1^ BA and 1.0 mg L^−1^ IBA (Declerk and Korban 1994). In other studies on this species, a high multiplication rate was obtained after the application of 1.0 mg L^−1^ BA. In turn, the presence of 0.13–0.25 mg L^−1^ TDZ in medium stimulated regeneration of numerous shoots but limited their elongation (Kaveriappa *et al.*^1997^). Similar results were obtained by Ďurkovič (2008) for *C. mas* ‘Macrocarpa’ on medium supplemented with 0.05–0.5 mg L^−1^ TDZ alone or in combination with either 0.5 mg L^−1^ BA or 0.05 mg L^−1^ NAA. However, these shoots were unable to root. This is unlike *C. alba*, where both cultivars produced a similar shoot number on media with 0.5–1.0 mg L^−1^ TDZ and 0.1 mg L^−1^ NAA and on media containing BA and NAA.

Normal shoot growth depends on the medium pH. For *C. florida* (Declerk and Korban 1994; Sharma *et al.*^2005^) and *Cornus* ‘NCCH1’ (Lattier *et al.*^2014^), a medium pH of 5.6–5.7 was used. Ďurkovič (2008) obtained the highest shoot number per explant of *C. mas* ‘Macrocarpa’ by increasing the medium pH to 6.2. A similar response to pH was observed in *C. alba* ‘Aurea’ and ‘Elegantissima’, where the highest shoot number was produced on medium with pH 5.8 and 6.2; however, at pH 6.8, the shoots were longer. According to Schubert *et al.* (1990), shoot elongation depends on ammonium nitrate and sucrose availability in the medium. Uptake of these compounds depends on medium acidity (Sakano 1990). During the culture period, explants take cations from the medium while organic acids leak out from their tissues, lowering the pH. Under low-pH conditions, protons (H^+^) pass from the cytoplasm into the intercellular spaces and in their place cations are taken up, especially NH_4_^+^. In turn, a high pH level stimulates release of OH^−^ and NO^3−^ adsorption (Schmitz and Lörz 1990).

In these trials with *C. alba*, concentrations of 20–30 g L^−1^ sucrose promoted regeneration as well as shoot number and length similarly in both cultivars. These observations are in accordance with results of Lech *et al.* (2009), who showed a similar growth of shoot cultures of *C. mas* ‘Titus’ and ‘Devin’ on a medium with sucrose or glucose. According to Borkowska *et al.* (1995), 20 g L^−1^ of sugars, regardless of their type, supplied the tissue demand for a source of energy and provided enough material to form biomass. An excess of sugar (usually provided as sucrose at a concentration of 30 g L^−1^) served to control osmotic pressure. Carbohydrates acted as signaling particles in all phases of plant development. Silva (2004) and Ahmad *et al.* (2007) indicated that sugars are perceived by the cell as chemical signals *in vitro*, with very high concentrations acting as stressing agents. Perata *et al.* (1997) reported that high sugar concentrations could inhibit gibberellin signaling and suppress cell division and growth in several different plant systems. Results of the present experiments are consistent with the above statements, as at high sucrose concentrations (40–50 g L^−1^) regeneration was poor and the shoots were shorter than those regenerated on lower sucrose concentrations (20–30 g L^−1^).

According to the literature, IBA positively affects rooting in most dogwood species. With *C. florida*, 52% of microcuttings rooted on a medium supplemented with 0.5 mg L^−1^ IBA, while 46% were rooted with 1 mg L^−1^ IBA (Kaveriappa *et al.*^1997^). For the same species, Sharma *et al.* (2005) obtained 83% rooted microshoots with well-developed root balls on medium containing 1.0 mg L^−1^ IBA. In addition, microcuttings of *C. canadensis* rooted quite well in the presence of 0.1 mg L^−1^ IBA in the rooting medium (Feng *et al.*^2009^). Ďurkovič and Bukovská (2009) reported quite different results with *C. mas* ‘Macrocarpa’, where 73% of microcuttings developed roots on medium enriched with 1.0 mg L^−1^ NAA. A similar response was observed here in both cultivars of *C. alba*, in which rhizogenesis was stimulated better by NAA than IBA. These findings differ from those of Zhang and Li (2005b, 2010), who reported that microshoots of *C. alba* generally rooted the best on medium with 0.05 mg L^−1^ IBA, while for ‘Aurea’ in particular the best root development was observed on medium with 0.5 mg L^−1^ IBA. In another study, microcuttings of *Cornus* ‘NCCH1’ rooted at a frequency of 72.5% on medium supplemented with 0.44 mg L^−1^ IAA (Lattier *et al.*^2014^). Similar results in different cultivars of *C. kousa* were obtained by Hadziabdic (2005), who used 0.1–2.4 mg L^−1^ IAA. This, however, contrasts with the findings of Konôpková and Bošiaková (2013), who rooted microcuttings of *C. kousa* on medium enriched with NAA, while no roots appeared in a treatment with IAA.

The final phase of micropropagation is acclimation of plantlets to greenhouse/field conditions. Plants of both white dogwood cultivars acclimated to *ex vitro* conditions at frequencies of 80–90%. These findings are consistent with those of Zhang and Li (2005b, 2010) with *C. alba* and those of Ďurkovič (2008) and Ďurkovič and Bukovská (2009) with *C. mas* ‘Macrocarpa’, which confirmed a high survival rate of acclimated plants (80%). A different result was obtained by Kaveriappa *et al.* (1997) with *C. florida*, in which only 60% of the plantlets survived the transfer to *ex vitro* conditions, probably due to dieback of apical buds and poor branching. On the other hand, Ishimaru *et al.* (1998) and Li *et al.* (2015) reported 100% survival of plantlets of *C. kousa* and *C. wilsoniana*, respectively.

Even when micropropagation can be achieved, it cannot be considered fully successful unless complete genetic fidelity is maintained. Various kinds of changes at the molecular level, such as single-nucleotide changes, deamplification and amplification of genes, alterations in DNA methylation patterns, and transposable element activations have been associated with genetic instability induced under *in vitro* conditions (Rani and Raina 2000). Ďurkovič and Bukovská (2009) and Ďurkovič (2009) did not observe any phenotypic and molecular changes of cornelian cherry ‘Macrocarpa’. Likewise, no somaclonal variation was observed with *C. wilsoniana* (Li *et al.*^2015^). Similar results were obtained here with *C. alba* ‘Aurea’ and ‘Elegantissima’, which were tested with both RAPD and ISSR markers, suggesting that no addition/deletion mutations occurred during the *in vitro* adventitious shoot organogenesis and acclimation period.

## Conclusions

The efficiency of micropropagation of *C. alba* ‘Aurea’ and ‘Elegantissima’ depends on macroelement composition, sucrose concentration, presence of growth regulators, and medium pH. Medium containing the WPM macronutrient complex, 20–30 g L^−1^ sucrose, BA, and NAA and adjusted to pH 6.2 stimulated shoot regeneration in both cultivars. White Dogwood microcuttings rooted better after supplementation of the medium with NAA than with IBA. Young plants of both cultivars, when transferred to *ex vitro* conditions, acclimated at a rate of 80–90% and did not show molecular changes relative to the mother plant. Therefore, the propagation method presented here may be applied commercially and as a basis for genetic engineering in white dogwood.
